# Paramagnetic Nanoparticles as a Platform for FRET-Based Sarcosine Picomolar Detection

**DOI:** 10.1038/srep08868

**Published:** 2015-03-09

**Authors:** Zbynek Heger, Natalia Cernei, Sona Krizkova, Michal Masarik, Pavel Kopel, Petr Hodek, Ondrej Zitka, Vojtech Adam, Rene Kizek

**Affiliations:** 1 Department of Chemistry and Biochemistry, Faculty of Agronomy, Mendel University in Brno, Zemedelska 1, CZ-613 00, Czech Republic, European Union; 2 Central European Institute of Technology, Brno University of Technology, Technicka 3058/10, CZ-616 00 Brno, Czech Republic, European Union; 3 Department of Pathological Physiology, Faculty of Medicine, Masaryk University, Kamenice 5, CZ-612 00 Brno, Czech Republic, European Union; 4 Department of Biochemistry, Faculty of Science, Charles University in Prague, Hlavova 2030, CZ-128 40 Prague 2, Czech Republic, European Union

## Abstract

Herein, we describe an ultrasensitive specific biosensing system for detection of sarcosine as a potential biomarker of prostate carcinoma based on Förster resonance energy transfer (FRET). The FRET biosensor employs anti-sarcosine antibodies immobilized on paramagnetic nanoparticles surface for specific antigen binding. Successful binding of sarcosine leads to assembly of a sandwich construct composed of anti-sarcosine antibodies keeping the Förster distance (*R*o) of FRET pair in required proximity. The detection is based on spectral overlap between gold-functionalized green fluorescent protein and antibodies@quantum dots bioconjugate (λ_ex_ 400 nm). The saturation curve of sarcosine based on FRET efficiency (F_604_/F_510_ ratio) was tested within linear dynamic range from 5 to 50 nM with detection limit down to 50 pM. Assembled biosensor was then successfully employed for sarcosine quantification in prostatic cell lines (PC3, 22Rv1, PNT1A), and urinary samples of prostate adenocarcinoma patients.

Prostate carcinoma (CaP) was determined to be the most common cause of cancer-related deaths in men in USA in 2013^1^. Early diagnostic of CaP is of great importance for successful elimination of metastatic expansion. Commonly used biomarker as prostate specific antigen (PSA) has been shown to have less sensitivity and specificity leading to both false-negative and/or false-positive results in some cases^2^,^3^, hence novel potential biomarkers have been searching. In 2009, sarcosine (Sar) was highlighted as a potential CaP biomarker enabling us to distinguish benign, localized or metastatic tumours with very high accuracy. This marker can be detected not only in blood, but also in urine, which allows a simple and non-invasive diagnostics^4^.

Gas chromatography mass spectrometry^4^ and liquid chromatography mass spectrometry^5^ are the most commonly utilized analytical methods for sarcosine determination. Using of sarcosine-oxidase as a part of (nano)biosensors allows specific determination of sarcosine by using photoelectrochemical^6^, fluorescent^7^ or colorimetric detection^8^ with limits of detection in nanomolar range. However, the accurate Sar identification in complex biological matrices as urine is still challenging, particularly its determination in the presence of other amino acids^9^. Thus, the development of novel and sensitive methods for precise determination of sarcosine is necessary for both confirmation of its potential to be used as valid CaP biomarker, and its application for screening purposes.

Herein we describe the method for sarcosine determination based on Förster resonance energy transfer (FRET) between quantum dots (QDs) and green fluorescent protein (GFP) enabled by sandwich arrangement using anti-sarcosine antibodies (Abs) in patient urinary samples and in prostatic cell lines (PC3, 22Rv1, PNT1A).

## Results and Discussion

### Characterization of the nanoconstruct

The superparamagnetic iron oxide nanoparticles (SPIONs - γ-Fe_2_O_3_ and Fe_3_O_4_) are versatile nanoscaled materials that have been applied in various fields of the interest^10^,^11^,^12^. They can be simply functionalized to provide binding sites towards various molecules and, moreover, they can be conveniently immobilized *via* external magnetic field. Hence, we exploited these nanostructures as a platform for FRET-based sarcosine biosensing.

**Figure 1:**
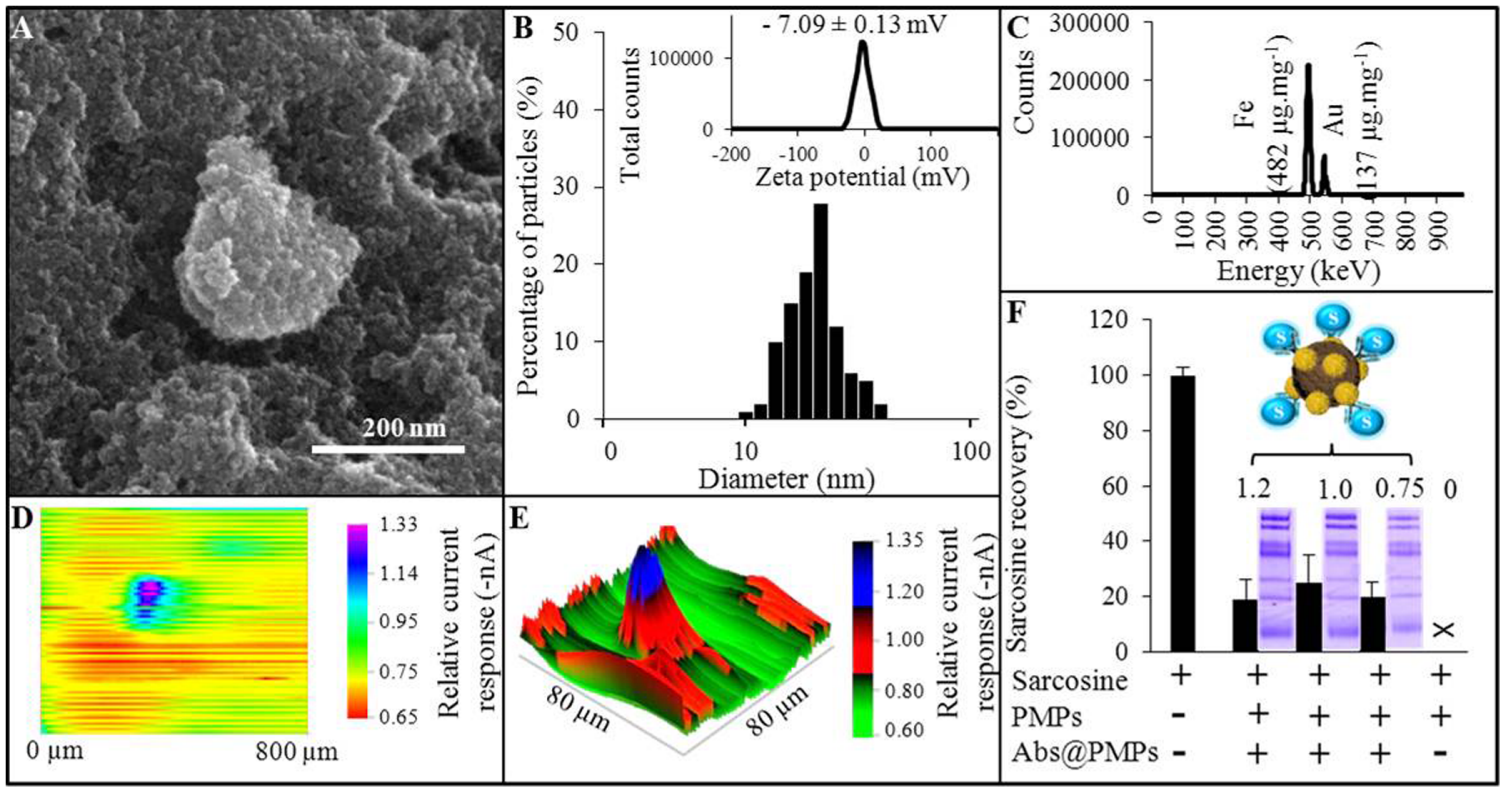
The characterization of paramagnetic microparticles, composed of nanomaghemite core, modified with polyvinylpyrrolidone and gold expressed as: (A) SEM micrograph (length of scale bar is 200 nm). (B) Particles size distribution, with expression of their zeta potential (determined in PBS, pH 7.4). (C) XRF spectrum showing the most abundant elements in paramagnetic particles. (D) SECM scans expressing the electrochemical current response behavior of immobilized nanoparticles (800 × 800 μm). (E) Scan was further converted to more detailed 3D scan (80 × 80 μm) that shows the decrease of relative current response influenced by nanoparticles presence. (F) Results of recoveries of sarcosine (2 μM) binding to antibodies at nanoparticles, obtained from IEC analyses. Values are means of three independent replicates (*n* = 3). Vertical bars indicate standard error. SDS-PAGE showing the binding capacity of nanoparticles towards sarcosine antibodies (0.75–1.2 mg.mL^−1^ of Abs) are illustrated too.

The morphology of prepared nanoparticles was determined by scanning electron microscopy. As it can be seen in Fig. 1A (scale bar 200 nm), nanoparticles form aggregates, due to their low zeta potential, determined as - 7.09 ± 0.13 mV (upper inset in Fig. 1B). Generally, iron oxide nanoparticles exhibit their absolute zeta potential within the pH range 4–10^13^, and thus using PBS (pH = 7.4) leads to aggregation of nanometric particles that was partially reduced using ultrasonic homogenizer (Bandelin electronic, Berlin, Germany) to form suspension containing particles with relatively uniform size (*d* = 23 ± 5 nm, Fig. 1B).

X-ray fluorescence spectra revealed that iron originating from nanomaghemite (γ-Fe_2_O_3_), which was used to constitute a paramagnetic core, formed almost one half of present elements (particularly 482 μg.mg^−1^). Gold, used for nanoparticles surface modification allowing their conjugation with antibodies or binding thiol-containing compounds, was identified as the second most abundant element (137 μg.mg^−1^, Fig. 1C).

Paramagnetic properties of the nanoparticles were also evidenced by SECM. It is shown in Figs. 1D–E that placement of a neodymium magnet under the detection electrode led nanoparticles attraction to one place, which was observed as local increasing of current response. Reduction of nanoparticles layer rapidly decreased the relative current response (from basic −0.70 nA to app. −1.33 nA).

To prepare sarcosine-sensing nanoparticles we employed anti-sarcosine antibodies isolated from egg yolks of sarcosine-immunized hens^14^. We examined the absorption of antibodies (electrostatic and/or hydrophobic interactions) on a surface of nanoparticles modified with gold. Primarily, the bioconjugation capacity was evaluated by SDS-PAGE of unbound Abs (original concentrations 0–10 mg.mL^−1^). The optimal coating of nanoparticles was achieved using antibodies dilution of 1–1.2 mg.mL^−1^ (Fig. 1F). For nanoparticles covered with 1 mg.mL^−1^ Abs, the recovery of sarcosine isolation from 2 μM standard solution was 25%, as calculated from determination of nanoparticles-attached sarcosine (Fig. 1F). Moreover, it was revealed that no sarcosine was bound to nanoparticles without Abs and therefore, there is no need to block their surface before their use for sarcosine isolation.

### FRET

**Figure 2:**
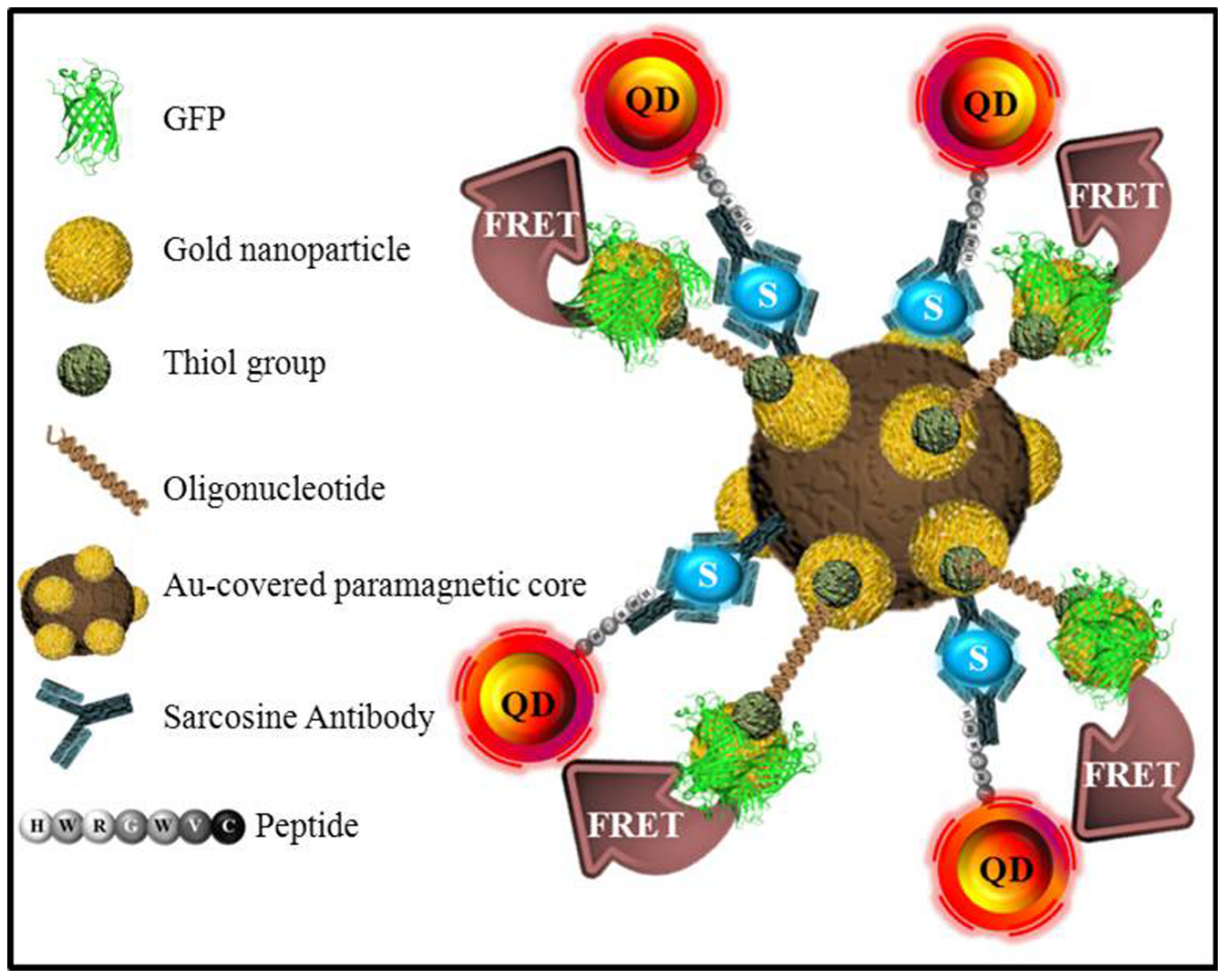
A schematic expression of FRET between green fluorescent proteins (green) and quantum dots (QD, red-yellow) on surface of paramagnetic nanoparticle modified with polyvinylpyrrolidone and gold. Spectral overlap is enabled by binding of sarcosine (S, blue) and provided by sandwich of its antibodies (all components shown in left part of figure).

The design of the suggested paramagnetic nanoparticles-based structure is schematically shown in Fig. 2. The main purpose of this biosensor is the isolation and detection of sarcosine with high specificity and sensitivity. By using preliminary ELISA experiments it was evaluated that sandwich assay with anti-sarcosine antibodies reached limit of detection of 8 nM for sarcosine (data not shown). Hence, we employed sarcosine as a linker, connecting two fluorophores to perform FRET, localized on paramagnetic nanoparticles.

Due to the fact that both, donor (GFP) and acceptor (QDs) have to be modified to enable specific binding to the target structures, we evaluated the possible changes of their fluorescence properties upon their modification. As a donor we employed GFP that was previously described to provide sufficient quantum yield for detection and moreover, it is sufficiently stable to be imaged during the experiment^15^. For GFP functionalization we utilized its modification with gold nanoparticles (AuNPs). Similarly to Bale *et al*.^16^ we employed a simple procedure comprising a mixing of GFP (in final concentration of 1 mg.mL^−1^) with AuNPs both in PBS.

**Figure 3:**
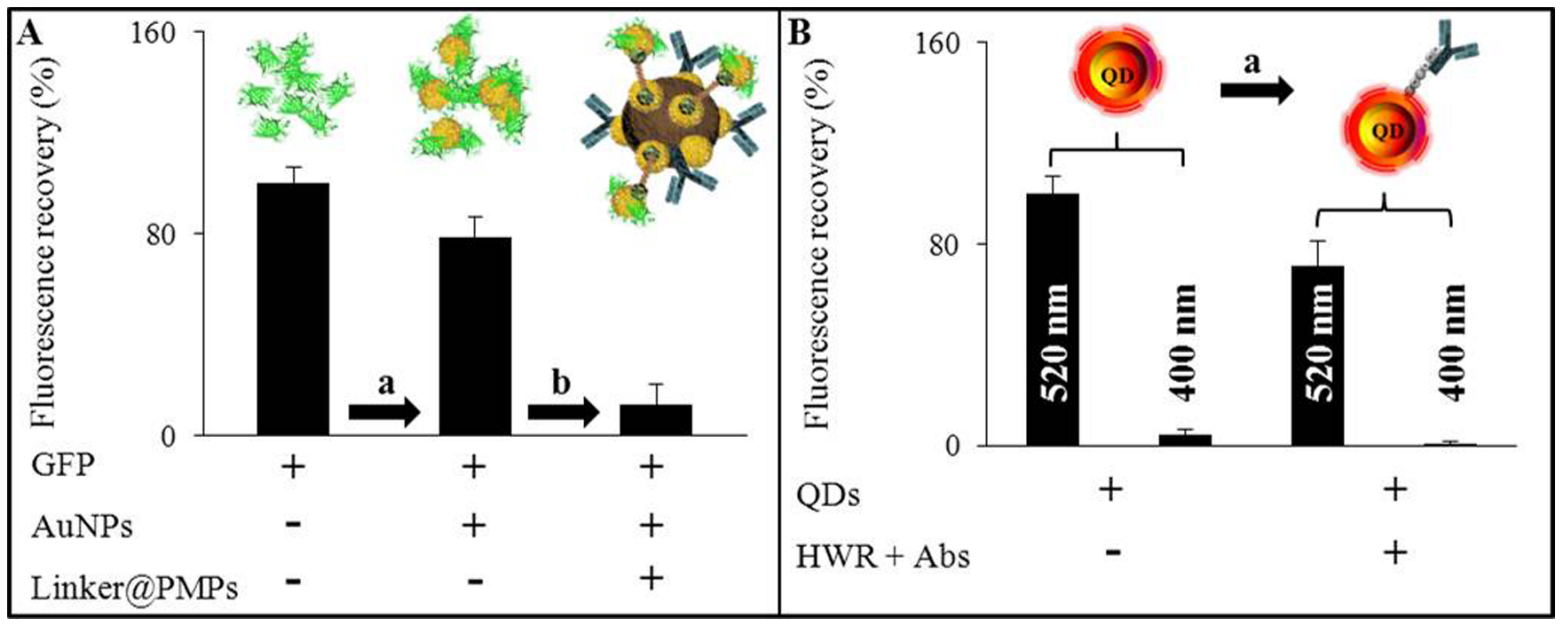
To provide the binding sites for oligonucleotide linker, GFP was modified with gold nanoparticles (AuNPs). During the whole workflow, (a) process of GFP conjugation and (b) subsequent binding to a surface on nanomaghemite through oligonucleotide linker, (A) fluorescence recovery was evaluated (λ_exc_ 400 nm and λ_em_ 510 nm). As the acceptors for fluorescent resonance transfer there were employed quantum dots with λ_exc_ 520 nm and λ_em_ 604 nm. (B) Their fluorescent properties were tested both in their optimal excitation and in the excitation of GFP (λ_exc_ 400 nm). (a) Fluorescence of bare QDs and fluorescence after their conjugation with HWR heptapeptide, carrying sarcosine antibodies was determined at λ_em_ 604 nm. Values are means of three independent replicates (*n* = 3). Vertical bars indicate standard error.

As it is shown in Fig. 3A, the modified GFP retained 78.9% of its fluorescence, indicating favorable retention of protein structure after functionalization. As gold binds thiols with high affinity and it does not undergo any unusual reactions with them^17^, we were able to use dsDNA with thiol moieties on both ends to form a linker between modified GFP and conjugate of Abs@PMPs. The resulting structure retained 12.3% of initial fluorescence of unmodified GFP exhibited paramagnetic properties and contained specific binding sites for sarcosine (Fig. 3A). Using atomic absorption spectrometry, the content of gold, originating from both protein functionalization and modification of nanoparticles, was determined as 0.0037 μg.mL^−1^.

QDs were shown to be one of the most suitable fluorophores in FRET configuration due to their exceptional brightness, high quantum yields and characteristic excitation and emission spectra^18^. A basic fluorescence characterization of our CdTe QDs revealed excitation maxima at λ_exc_ 520 nm with emission peak at λ_em_ 604 nm. Using excitation of GFP (λ_exc_ 400 nm), very weak fluorescence was obtained (4.2% when compared with 100%, determined in QDs excitation maxima, Fig. 3B). To obtain the acceptor molecules with the required parameters as high fluorescence and specificity, we employed peptide to connect QDs with anti-sarcosine antibodies. Synthetic heptapeptide HWRGWVC was previously exploited for bioconjugation of QDs with human immunoglobulin by us^19^. Hence, we employed the natural ability of its *N*-terminus composed of histidine followed by aromatic and positively charged amino acids to recognize and bind antibodies through their Fc region. The major advantage is that peptide-mediated conjugation controls the orientation of antibody to face the antigen binding site outward. Moreover, cysteine at the peptide *C*-terminus serves as a stabilization agent of colloidal nature of CdTe QDs solution. As it can be seen in Fig. 3B, the conjugation resulted in decrease of fluorescence of QDs to 71.3% of initial value while maintaining their excitation and emission maxima (λ_exc_ 520 nm; λ_em_ 604 nm).

Close contact of two fluorophores and their spectral overlap allows the desired Förster/fluorescence resonance emission transfer. We mixed together both donor (GFP connected to paramagnetic nanoparticle bearing Abs with bound sarcosine), and acceptor (Abs@QDs conjugate). After the incubation (37°C, 800 rpm, 1 h) the surplus of liquid was removed while complex was immobilized in vial using external magnetic field and washed with PBS with 0.5% Tween-20 and 1% bovine serum albumin (BSA), which protects QDs against quenching, caused by induction of their colloidal instability, resulting from interaction with buffer components. The size of assembled biosensor was established to be relatively uniform within 3 different batches (*d* = 103 ± 11 nm) with standard deviation of 6%. Further characterization revealed that GFP content in assembled biosensor was app. 224 μg.mL^−1^, while the level of the major component of QDs as cadmium was found to be 58 μg.mL^−1^. Based on these fact and protocol published by Casanova et al.^20^, we found that the number of FRET pair(s) per nanoparticle was 0.25. which is cause mainly due to the excess of the particles to GFP and QDs. Finally, a fluorescence behavior was studied.

### Detection of sarcosine

**Figure 4:**
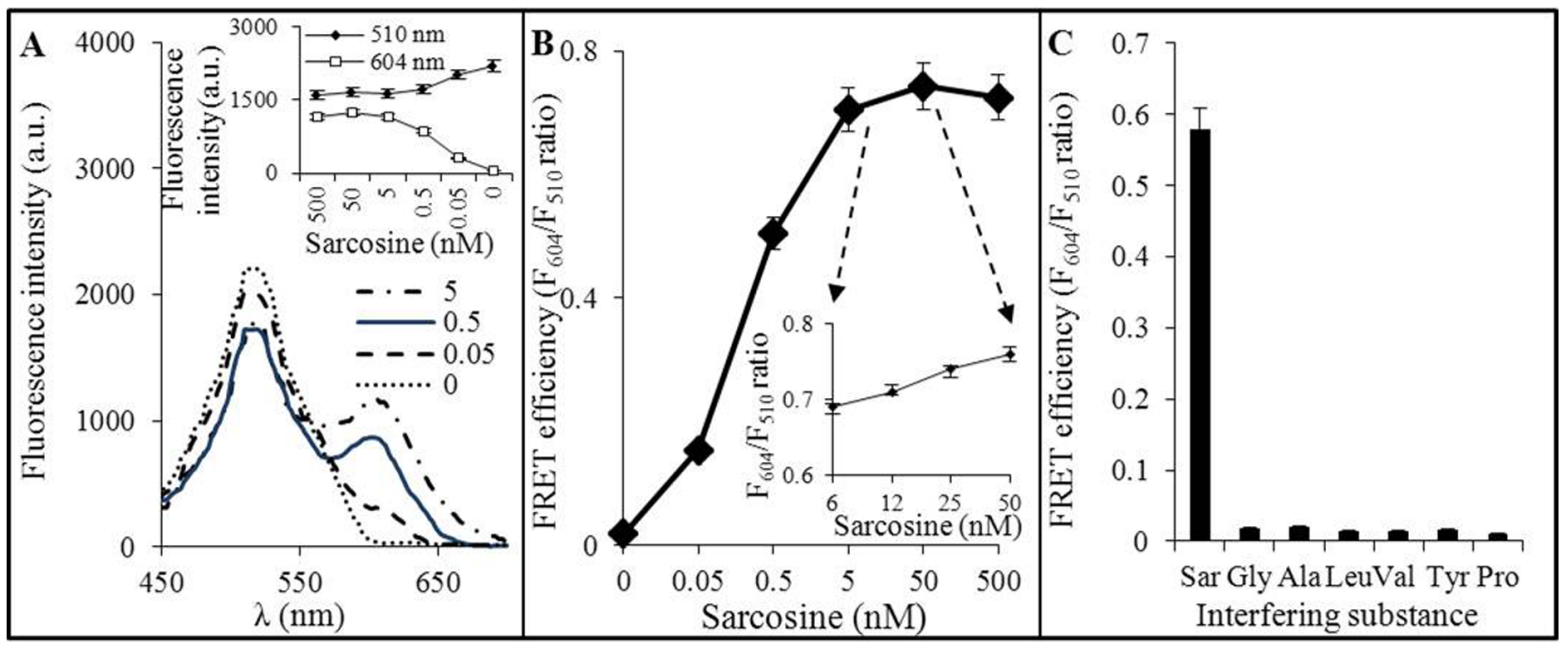
(A) Fluorescence emission spectra of FRET were recorded for 0–5 nM sarcosine at 25°C. The excitation wavelength was set to 400 nm. In inset: there is the expression of fluorescence (λ_exc_ 400 nm) dependence on sarcosine concentration evaluated in both emission maxima of GFP (λ_em_ 510 nm) and QDs (λ_em_ 604 nm). (B) Saturation curve of sarcosine (0–500 nM, diluted in PBS, pH 7.4). In inset: the calibration curve determined within linear range 5–50 nM. (C) The expression of interfering potential of various amino acids (all of them diluted in PBS, pH 7.4 to the final 50 nM concentrations) on FRET efficiency. Values are means of three independent replicates (*n* = 3). Vertical bars indicate standard error.

Sarcosine was shown to be crucial for FRET, while its absence led to detection of donor fluorescence (λ_em_ 510 nm) only with no effect on FRET (Fig. 4A). Importantly, it was confirmed that the acceptor (Abs@QDs conjugate) has no ability to join the paramagnetic complex *via* an unspecific bond. In the case of sarcosine captured in antibodies sandwich, it was observed that donor excitation triggers spectral overlap leading to “lighting up” of the acceptor. Hence, it was shown that our construct can assemble a spatial orientation enabling a FRET and moreover, it was revealed that FRET efficiency (F_604_/F_510_ ratio, which describes the relationship between an increase of acceptor fluorescence intensity at the expense of the fluorescence intensity of donor upon its excitation) is dependent on antigen concentration. As it can be seen in inset in Fig. 4A, the acceptor emission intensity is proportional to concentration of antigen and spectral overlap is observed till 50 pM sarcosine, which is more sensitive than other described biosensors^6^,^7^,^8^. This phenomenon is caused by saturation of certain number of antibodies, resulting in propagation of sandwich binding sites on sarcosine molecule towards limited amount of acceptors. On the other hand, using concentrations higher than 50 nM, the antibodies reach saturation plateau disallowing quantification of antigen. Determination of sarcosine was tested within linear dynamic range (5–50 nM) of the saturation curve (Fig. 4B), based on FRET efficiency (F_604_/F_510_ ratio), with the parameters of y = 0.024x + 0.665, R^2^ = 0.993). Due to the fact that sarcosine amount in clinical samples of the prostatic onco-patients varies widely; sarcosine quantification is fundamentally dependent on a proper dilution of analyzed sample. The accomplishment of this requirement leads to very sensitive and specific detection.

As mentioned above, sarcosine analyses are difficult due to presence of many interfering compounds complicating the most commonly used analyses separation (GC/LC) hyphenated to mass detection^9^. The amino acids commonly present in urine were tested for their interfering potential (Fig. 4C). It was revealed that our sensor is able to distinguish sarcosine in the presence of various urinary amino acids including alanine. Negligible FRET efficiency was determined as a result of a background fluorescence of solution (F_604_/F_510_ ratio 0.01–0.02 compared with 0.58 of sarcosine).

**Figure 5:**
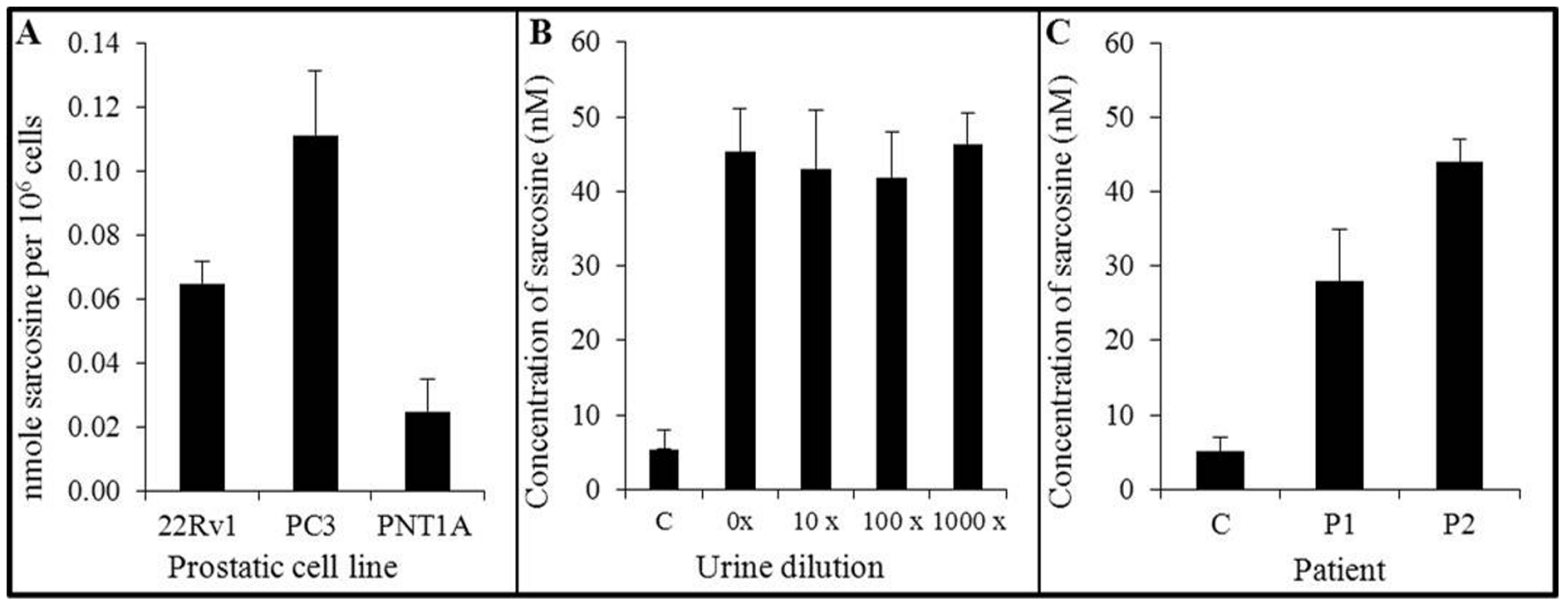
An utilization of antibodies for sarcosine isolation from the real samples of urine. (A) Sarcosine amount isolated from prostatic cell lines (PC3, 22Rv1, PNT1A). (B) Urinary samples were spiked with 40 nM sarcosine and various dilution were tested (0×; 10×; 100×; and 1000×) and compared to control (C) without sarcosine spike. Analyses were performed using FRET at λ_exc_ 400 nm and concentrations were calculated according to calibration curve. (C) Sarcosine concentration determined in real samples of patients suffering from acinar adenocarcinoma of prostate. The urinary samples were diluted 100× prior to isolation. Values are means of three independent replicates (*n* = 3) analyzed with different batches of system. Vertical bars indicate standard error.

Based on obtained results we hypothesized that our approach can be applicable also for evaluation of sarcosine levels in prostatic cell lines and clinical urinary samples. Firstly, Abs@PMPs conjugate was employed to quantify sarcosine in disrupted prostatic cells. It was shown that sarcosine is present in all types of prostatic cells, including normal prostatic epithelial cells (PNT1A) - 0.03 nmol per 10^6^ cells, human carcinoma epithelial cells derived from a xenograft propagated in mice (22Rv1) - 0.07 nmol per 10^6^ cells, and human cell line from androgen independent prostatic adenocarcinoma derived from metastatic site in bone (PC3) - 0.11 nmol per 10^6^ cells (Fig. 5A). These results are in agreement with results published by Khan and colleagues used glycine-*N*-methyl transferase (GNMT) knockdown cell lines for sarcosine assessment^21^.

The primary purpose of our sarcosine sensor was the isolation and quantification of urinary sarcosine as the non-invasive biomarker. Urine is one of the most accessible and stable body fluids however, it is rather complex biological matrix containing interfering compounds, which may reduce the effectiveness of analytical assays^22^. Hence, to observe the influence of this matrix, we diluted control urinary sample (0×; 10×; 100×; and 1000×) and all of diluted aliquots were spiked with 40 nM sarcosine. Sarcosine (5.4 nM) was determined also in control non-spiked samples (Fig. 5B). Furthermore, it was revealed that the urine dilution does not influence sarcosine binding to Abs and all of the diluted aliquots show comparable content of antigen determined *via* FRET (41.9–46.3 nM).

Presented data allow us to proceed with real clinical samples obtained from prostate adenocarcinoma patients. Regarding to results obtained using IEC we diluted two analyzed urinary samples 100× with water. For comparison one undiluted control was employed too (Fig. 5C). The use of Abs@PMPs conjugate and subsequent assembly of entire FRET construct resulted in sarcosine identification in both urinary samples. FRET quantification of antigen in 100× diluted urine showed that the content of the target analyte was 28 and 44 nM in sample 1 and 2. If we calculate the dilution and compare the results with those found by IEC (3.3 and 5.2 μM), we can conclude that these methods are in very good agreement. Moreover, three different batches of FRET system were tested with standard deviation of 2–7%. Hence, it can be stated that isolation of sarcosine through Abs followed by FRET analysis may provide very specific and sensitive bioanalytical approach. Particularly, the sensitive determination of sarcosine changes during the disease progression may provide useful prognostic and diagnostic tool able to increase the successful diagnostic rate of prostate carcinoma^23^, leading to decrease of mortality associated with this disease.

In conclusion, we successfully synthesized nanomaghemite core-based paramagnetic nanoparticles, containing the binding sites for sarcosine antibodies and thiol moieties of oligonucleotide linker, bearing GFP functionalized with AuNPs. Abs@PMPs conjugate was able to bind sarcosine in biological samples as prostatic cell lines and urinary samples of onco-patients. Using HWR heptapeptide, Abs@QDs acceptor molecule was constructed and using sandwich binding of antibodies FRET was observed, and this was dependent on sarcosine concentration. We were able to discriminate sarcosine in both prostatic cells and urinary samples with very good sensitivity and without undesired interference. Our approach may be exploited for quantification of sarcosine fluctuations in time for prognostic approaches.

## Methods

### Chemicals

All reagents for syntheses, native oligonucleotides, standards, and other chemicals were purchased from Sigma-Aldrich (St. Louis, MO, USA) in ACS purity, unless noted otherwise.

### Synthesis of nanomaghemite, gold nanoparticles, quantum dots and HWR peptide

Nanometric maghemite was prepared by NaBH_4_ reduction of FeCl_3_·6H_2_O according to protocol as follows: 1 g of FeCl_3_·6 H_2_O (3.7 mM) was dissolved in 80 mL of MilliQ water and a solution of 0.2 g of NaBH_4_ (5.3 mM) in ammonia (3.5%, 10 mL) was poured into the first solution under vigorous stirring. The obtained solution was boiled for 2 h. After cooling down the magnetic nanoparticles were separated by external magnetic field and washed with water for several times. Subsequently, nanomaghemite particles were suspended in water solution of 20 mL of 7.5% polyvinylpyrrolidone (PVP) (40 μM) and modified with 1 mM HAuCl_4_, followed by addition of 0.75 mL of 0.1 M trisodium citrate. The resulting mixture was stirred overnight, separated using an external magnetic force field and dried at 40°C.

Gold nanoparticles were prepared by "citrate" method. Aqueous solution of sodium citrate (0.5 mL, 40 mM) was added to a solution of HAuCl_4_·3 H_2_O (10 mL, 1 mM). The mixture was stirred overnight.

CdTe quantum dots were prepared by mixing of 10 mL of 20 mM solution of cadmium acetate dihydrate in MiliQ water (76 mL) with 1 mL of 4 mM mercaptosuccinic acid dissolved in water, followed by addition of 1.8 mL of 1 M NH_3_. Finally, 1.5 mL of 10 mM solution of Na_2_TeO_3_ was added and after few minutes 50 mg of NaBH_4_ (1.3 mM) was poured to the stirred solution. After 1 hour stirring, the volume of reaction mixture was adjusted to 100 mL using water and the solution was heated in microwave reactor Multiwave 300 (Anton Paar, Graz, Austria) at 300 W and 120°C for 10 min.

The heptapeptide with the amino acid sequence HWRGWVC (943.0912 Da) was prepared on Liberty Blue peptide synthesizer (CEM, Matthews, NC, USA) by standard Fmoc solid-phase synthesis using 20% piperidine in dimethylformamide (*v/v*). Purity of crude peptide was analysed using high performance liquid chromatography with UV detection (ESA Inc., Chelmsford, MA, USA) and matrix-assisted laser ionization/desorption time-of-flight mass spectrometry (Bruker ultrafleXtreme, Bruker Daltonik GmbH, Germany).

### Characterization of modified nanometric maghemite particles

Firstly, the morphology of nanoparticles was observed using scanning electron microscope MIRA3 LMU (Tescan, a.s., Brno, Czech Republic). An accelerating voltage of 15 kV and beam current about 1 nA were used with satisfactory results regarding to maximum throughput.

Particles size and zeta potential was measured by Particle Size Analyzer (Zetasizer Nano ZS90, Malvern instruments, Malvern, UK). For this purpose, particles were dispersed in phosphate buffered saline (PBS, 137 mM NaCl, 2.7 mM KCl, 1.4 mM NaH_2_PO_4_, and 4.3 mM Na_2_HPO_4_, pH 7.4) and incubated at 25°C for 15 min prior the measurement.

Elemental composition was evaluated using X-ray fluorescence spectrometer Xepos (SPECTRO GmbH, Kleve, Germany), with parameters set as follows - measurement duration: 300 seconds, tube voltage from 24.81 to 47.72 kV, tube current from 0.55 to 1.0 mA.

Scanning electrochemical microscopy was employed to obtain data on electrochemical behavior of nanoparticles layer. Microscope Model 920D (CH instruments, Inc., Austin, TX, USA) consisted of 10 mm measuring platinum disc probe electrode with potential of 0.2 V. The measurement was performed in Teflon cell with volume of 1.5 mL under the following setting: amperometric mode, vertical scan was carried out in the area 500 × 500 μm with rate of 30 μm.s^−1^.

Binding capacity of nanoparticles was determined using sodium dodecyl sulfate polyacrylamide gel electrophoresis (SDS-PAGE) in 12.5% (*w*/*w*) separation gel and 5% (*w*/*w*) focusing gel. Separation was conducted at 120 V for 80 min.

### Production of sarcosine antibodies

Leghorn hens were immunized weekly by three subcutaneous injections (0.1 mg/dose/animal) with Sar immunogen (Sar-beta-Ala-beta-Ala-Cys-NH_2_) conjugated to maleimide-activated keyhole limpet haemocyanin (KLH) (Imject Maleimide-Activated mcKLH, Thermo Scientific, Rockford, IL, USA). The conjugation of hapten to carrier protein (KLH) was performed according to manufacturer's instructions. Preparation of antibodies and their immunoreactivity tests were carried out according to previously optimized protocol^14^. The entire process was conducted in accordance with the Regulations for the Care and Use of Laboratory Animals (311/1997, Ministry of Agriculture, Czech Republic).

### Bioconjugation of antibodies on paramagnetic nanoparticles and isolation of sarcosine

Prior to a bioconjugation, paramagnetic nanoparticles were washed with PBS to remove impurities. For bioconjugation, sarcosine antibodies were mixed with paramagnetic nanoparticles (0.5 mg.100 μL^−1^) in ratio 1:1. The resulting mixture was incubated for 60 minutes under shaking (800 rpm) at 37°C in thermoblock ThermomixerR (Eppendorf AG, Hamburg, Germany). After the incubation, the conjugate of antibodies at nanoparticles (Abs@PMPs) was washed three times with PBS and prepared for sarcosine binding. The binding of Sar with Abs@PMPs was carried out simply through the addition of conjugate to a sample (standard, urine, and/or cell lines), containing Sar. The conditions of isolation were as follows: 60 min, 800 rpm, 37°C, thermoblock ThermomixerR (Eppendorf AG, Hamburg, Germany). The bioconjugate with bound sarcosine was removed using an external magnetic field and three times washed with PBS and stored for further experiments.

### Preparation of donor - green fluorescent protein functionalized with AuNPs

For *Escherichia coli* transformation, the pGLO plasmid (Bio-Rad, Philadelphia, PA, USA), containing the reporter gene for green-fluorescent protein (GFP) was employed. *GFP* gene was under control of the arabinose-induced promoter araBAD and the *araC* gene encoding regulator protein. Bacteria transformed with pGLO plasmid were selected according to their ampicillin resistance. The positive transformants were grown in Luria-Bertani broth with 100 mg.L^−1^ of ampicillin and 0.2% arabinose (*w*/*w*) and incubated at 32°C overnight. The cells were harvested using centrifuge type 5340R (Eppendorf AG, Hamburg, Germany) at 4000 rpm for 10 min. The pellets were resuspended using TE buffer (1 mM EDTA with pH 8, 10 mM TRIS-HCl pH 7.5). To initiate the enzymatic digestion of the bacterial cell wall 10 mg.mL^−1^ of fresh lysozyme was added. The protein fractions were collected by centrifugation for 10 minutes at 14 000 rpm and 4°C. GFP was further purified from the bacterial lysate using fast protein liquid chromatography system Biologic DuoFlow (Bio-Rad, Philadelphia, PA, USA) and gel filtration column (Macro-Prep Methyl HIC support, Bio-Rad, Philadelphia, PA, USA). GFP separation was done by isocratic elution with ammonium sulfate. The presence of GFP was confirmed using matrix-assisted laser desorption/ionization time-of-flight mass spectrometry (MALDI-TOF MS) Bruker Ultraflextreme (Bruker Daltonik GmbH, Bremen, Germany) and SDS-PAGE. After the purification, GFP was functionalized with gold nanoparticles (AuNPs) through simple attachment of protein (1 mg.mL^−1^) on AuNPs dissolved in PBS. The suspension was shaken overnight at 25°C and the resulting one was filtered through Amicon Ultra Centrifugal Filters with ultracel-3 membrane (Merck Millipore, Darmstadt, Germany) to remove the excess of unbound AuNPs.

### Atomic absorption spectrometry

Gold in GFP and cadmium originating from QDs was determined on 280Z Agilent Technologies AAS (Agilent, Santa Clara, CA, USA) with electrothermal atomization. As the radiation source gold/cadmium ultrasensitive hollow cathode lamp (lamp current of 4 mA) was employed. The spectrometer was operated at 242.8/228.8 nm resonance line with spectral bandwidth of 1.0/0.5 nm. The pyrolysis temperature 500°C for 8 s and the atomization temperature 2600°C for 3 s were applied. The flow of argon was 300 mL.min^−1^. Zeeman background correction was used with field strength of 0.8 T. Cadmium was determined in the presence of palladium (1 g.L^−1^) chemical modifier.

### Preparation of acceptor – quantum dot-heptapeptide-antibody conjugate

To control the proper orientation of antibodies localized on acceptor molecule (QDs), the synthetic heptapeptide was employed as a linker between Abs and QD according to our previous study^19^.

### Prostatic cell lines types and growth conditions

Three human prostatic cell lines purchased from Health Protection Agency Culture Collection (Salisbury, UK) were used for experiments as follows: i) PNT1A established by immortalization of normal adult prostatic epithelial cells by transfection with a plasmid containing SV40 genome with a defective replication origin, ii) 22Rv1 a human prostate carcinoma epithelial cells derived from a xenograft serially propagated in mice after castration-induced regression and relapse of the parental, androgen-dependent CWR22 xenograft, and iii) PC-3 human cell line established from a grade 4 androgen independent and unresponsive prostatic adenocarcinoma obtained from 62-year-old Caucasian male and derived from metastatic site in bone. PNT1A and 22Rv1 were cultured in RPMI-1650 medium with 10% fetal bovine serum (FBS). PC-3 cells were cultured in Ham's F12 medium with 7% FBS. The media were supplemented with penicillin (100 U.mL^−1^) and streptomycin (0.1 mg.mL^−1^) and the cells were maintained at 37°C in a humidified incubator (Sanyo, Japan) with 5% CO_2_.

### Preparation of prostatic cell lines prior to sarcosine isolation

The prostatic cells were harvested and washed with PBS. Prior to isolation the cells were frozen by liquid nitrogen to disrupt their structure. The frozen sample was further homogenized using ultrasonic homogenizer SONOPLUS mini20 (Bandelin electronic, Berlin, Germany). Then, 1 mL of 0.2 M phosphate buffer (pH = 7.0) was added and the sample was homogenized for 5 minutes. The cell homogenates were then treated with Abs@PMPs bioconjugate.

### Real urinary samples

To determine the interference of substances occurred in urine, real samples of urine were diluted (0×, 10×, 100×, 1000×) and spiked with sarcosine to final concentration of 40 nM. These samples (250 μL) were incubated with Abs@PMPs (0.5 mg) under the following: 60 min, 800 rpm and 37°C. Particles were removed from urine using external magnetic field (Chemagen, Baesweiler, Germany) and Abs@PMPs with bound sarcosine was processed for FRET assay. Sarcosine was also isolated from urinary samples of patients suffering from acinar adenocarcinoma of prostate (obtained from St. Anne's University Hospital, Department of Urology in Brno). Detailed information about patients is shown in Table 1. Enrolment of patients into realized clinical study was approved by the Ethic committee of the Faculty of medicine, Masaryk University, Brno, Czech Republic. As a control sample, urinary samples obtained from healthy individual (25 years) were used. Informed consent was obtained from all subjects, healthy and patient ones.

### Sarcosine determination using ion-exchange liquid chromatography

For determination of sarcosine an ion-exchange liquid chromatography (Model AAA-400, Ingos, Prague, Czech Republic) with post column derivatization by ninhydrin and absorbance detector in visible light range (VIS) was used. Glass column with inner diameter of 3.7 mm and 350 mm length was filled manually with strong cation exchanger (Ostion LG ANB, Ingos, Prague, Czech Republic) 12 μm particles, 8% porosity in sodium cycle. Double channel VIS detector with inner cell of 5 μL was set to two wavelengths: 440 and 570 nm. Recovery of sarcosine was determined according to our previous study^24^.

### Fluorescence measurements

Fluorescence analyses were performed using multifunctional microplate reader Tecan Infinite 200 PRO (TECAN, Maennedorf, Switzerland). Samples (50 μL) were applied into UV-transparent 96 well microplate with flat bottom Costar® (Corning, NY, USA). All measurements were performed at 25°C controlled by Tecan Infinite 200 PRO. Fluorescence attributes were evaluated at two excitations (λ_exc_ 400 nm for GFP and λ_exc_ 520 nm for QDs) and two emission wavelengths (λ_em_ 510 nm for GFP and λ_em_ 604 nm for QDs).

## Author Contributions

Z.H. participated on FRET measurements and prepared the draft of the manuscript. N.C. performed the chromatographic detection of sarcosine and processed and discussed the results. S.K. performed antibodies based experiments and participated on the preparation of the nano-construct. M.M. performed the cell experiments and prepared cell and urinary samples prior to analysis. P.K. synthesized quantum dots and particles. P.H. prepared the antibodies and participated on the preparation of the nano-construct. O.Z. participated in the preparation of the manuscript. V.A. helped with the results discussion and conceived the study. R.K. designed experiments, discussed the results and submitted the manuscript.

## Figures and Tables

**Table 1:** Overview of the information of the acinar adenocarcinoma patients

Patient	PSA*	fPSA**	cT***	pT****	GS*****
**1**	7.07	0.22	T1c	pT2c	3 + 4
**2**	8.78	-	T2c	pT3a	3 + 3

*Prostate specific antigen (ng.mL^−1^), **free prostate specific antigen (free/total PSA ratio), ***clinical stage: *T1c* - tumor identified by needle biopsy, PSA elevated, *T2c* – both lobes affected. ****Pathological stage: *pT2c* - tumor affects both lobes, *pT3a* - tumor extends beyond the prostate. *****Gleason score.
